# Mass Treatment with Azithromycin for Trachoma: When Is One Round Enough? Results from the PRET Trial in The Gambia

**DOI:** 10.1371/journal.pntd.0002115

**Published:** 2013-06-13

**Authors:** Emma M. Harding-Esch, Ansumana Sillah, Tansy Edwards, Sarah E. Burr, John D. Hart, Hassan Joof, Mass Laye, Pateh Makalo, Ahmed Manjang, Sandra Molina, Isatou Sarr-Sissoho, Thomas C. Quinn, Tom Lietman, Martin J. Holland, David Mabey, Sheila K. West, Robin Bailey, on behalf of the Partnership for Rapid Elimination of Trachoma (PRET) study group

**Affiliations:** 1 London School of Hygiene & Tropical Medicine, London, United Kingdom; 2 National Eye Health Programme of The Gambia, Kanifing, The Gambia; 3 Medical Research Council Unit, Fajara, The Gambia; 4 National Institute for Allergy and Infectious Diseases, Bethesda, Maryland, United States of America; 5 University of California, San Francisco, California, United States of America; 6 Dana Center for Preventive Ophthalmology, Johns Hopkins University, Baltimore, Maryland, United States of America; University of California San Diego School of Medicine, United States of America

## Abstract

**Background:**

The World Health Organization has recommended three rounds of mass drug administration (MDA) with antibiotics in districts where the prevalence of follicular trachoma (TF) is ≥10% in children aged 1–9 years, with treatment coverage of at least 80%. For districts at 5–10% TF prevalence it was recommended that TF be assessed in 1–9 year olds in each community within the district, with three rounds of MDA provided to any community where TF≥10%. Worldwide, over 40 million people live in districts whose TF prevalence is estimated to be between 5 and 10%. The best way to treat these districts, and the optimum role of testing for infection in deciding whether to initiate or discontinue MDA, are unknown.

**Methods:**

In a community randomized trial with a factorial design, we randomly assigned 48 communities in four Gambian districts, in which the prevalence of trachoma was known or suspected to be above 10%, to receive annual mass treatment with expected coverage of 80–89% (“Standard”), or to receive an additional visit in an attempt to achieve coverage of 90% or more (“Enhanced”). The same 48 communities were randomised to receive mass treatment annually for three years (“3×”), or to have treatment discontinued if *Chlamydia trachomatis (Ct)* infection was not detected in a sample of children in the community after mass treatment (stopping rule(“SR”)). Primary outcomes were the prevalence of TF and of *Ct* infection in 0–5 year olds at 36 months.

**Results:**

The baseline prevalence of TF and of *Ct* infection in the target communities was 6.5% and 0.8% respectively. At 36 months the prevalence of TF was 2.8%, and that of *Ct* infection was 0.5%. No differences were found between the arms in TF or *Ct* infection prevalence either at baseline (Standard-3×: TF 5.6%, *Ct* 0.7%; Standard-SR: TF 6.1%, *Ct* 0.2%; Enhanced-3×: TF 7.4%, *Ct* 0.9%; and Enhanced-SR: TF 6.2%, *Ct* 1.2%); or at 36 months (Standard-3×: TF 2.3%, *Ct* 1.0%; Standard-SR TF 2.5%, *Ct* 0.2%; Enhanced-3× TF 3.0%, *Ct* 0.2%; and Enhanced-SR TF 3.2%, *Ct* 0.7% ). The implementation of the stopping rule led to treatment stopping after one round of MDA in all communities in both SR arms. Mean treatment coverage of children aged 0–9 in communities randomised to standard treatment was 87.7% at baseline and 84.8% and 88.8% at one and two years, respectively. Mean coverage of children in communities randomized to enhanced treatment was 90.0% at baseline and 94.2% and 93.8% at one and two years, respectively. There was no evidence of any difference in TF or *Ct* prevalence at 36 months resulting from enhanced coverage or from one round of MDA compared to three.

**Conclusions:**

The Gambia is close to the elimination target for active trachoma. In districts prioritised for three MDA rounds, one round of MDA reduced active trachoma to low levels and *Ct* infection was not detectable in any community. There was no additional benefit to giving two further rounds of MDA. Programmes could save scarce resources by determining when to initiate or to discontinue MDA based on testing for *Ct* infection, and one round of MDA may be all that is necessary in some settings to reduce TF below the elimination threshold.

## Author Summary

Trachoma, which results from infection with a bacterium *Chlamydia trachomatis(Ct)*, is a leading cause of preventable blindness in the world. One of the currently used control methods is mass drug administration (MDA) with azithromycin, which is initiated according to rates of follicular trachoma(TF) in children. This study was a clinical trial done to determine whether testing communities for *Ct* infection will prevent unnecessary rounds of MDA. This was done by allowing communities to stop treatment if their infection had been reduced below a threshold. The study compared the effects of one round of mass treatment to three and found that there was no difference in either follicular trachoma or infection rates after three years. One round of treatment reduced TF to a low level. Tests for infection could be used to decide when to start or discontinue MDA and to prevent unnecessary treatment rounds in settings like The Gambia.

## Introduction

Trachoma, caused by repeated ocular infection with the bacterium *Chlamydia trachomatis (Ct)*, is the leading infectious cause of blindness worldwide [1]. There are an estimated 325 million people living in trachoma endemic areas, 31 million people with active trachoma (trachomatous inflammation, follicular (TF) and/or trachomatous inflammation, intense (TI)) and 7 million suffering from trichiasis [2]. The World Health Organization (WHO) recommended trachoma control strategy, known by the acronym SAFE, has as its components **S**urgery for trichiasis, **A**ntibiotic treatment of affected communities, and the promotion of **F**acial cleanliness and **E**nvironmental improvement.

In 1998, the World Health Assembly called on countries to determine the prevalence of trachoma and to implement the SAFE strategy [3]. Annual mass drug administration (MDA) with azithromycin (or topical tetracycline) for three years, was recommended by WHO, in combination with other elements of the SAFE strategy, in districts where the prevalence of TF in children aged 1–9 years, determined by programmes using population–based surveys, was at least 10%. The WHO's manual for trachoma programme managers recommended that where the district prevalence was between 5 and 10% in 1–9 year olds TF prevalence be assessed at community level and MDA initiated in any community where the prevalence in 1–9 year olds was 10% or greater. This recommendation has, however, not been widely implemented. A further recommendation was that, once treatment was initiated in a community, re-assessment of the need to continue or stop it was unnecessary before three years [4]. More than 280 million doses of the antibiotic azithromycin have been donated for MDAs by Pfizer via the International Trachoma Initiative (ITI) since 1999, in 19 trachoma-endemic countries with a national trachoma control plan [5].

The Gambia is in the arid Sahel belt of West Africa where trachoma has probably been endemic for centuries. However, national surveys carried out in 1986 and 1996 showed a falling prevalence of trachoma, which was the second leading cause of blindness in 1986 (17% of all blindness), but only the fifth leading cause in 1996 (5.6% of all blindness). Over the same interval the national prevalence of active trachoma in 0–14 year-olds fell from 10.4% to 4.9%.[6] The Gambian National Eye Health Programme (NEHP), established in 1986, expanded its national intervention programme to cover the whole country by 1996. Prior to constructing a national trachoma control plan involving donated azithromycin, The Gambia was asked by the ITI technical expert committee to re-survey two of its affected regions to re-establish trachoma prevalence data. This survey, which was conducted in The North Bank and Lower River regions in 2006 found that the overall prevalence of TF in children aged 1–9 years in the two regions was 10.7%, and not much changed from 1996. However, there was a large and geographically widespread discrepancy between the prevalence of TF and the presence of ocular *Ct* infection, which was found in only 0.3% of the children tested [7]. In the 2007 trachoma control plan [8] The Gambian NEHP used this data to prioritise eleven districts known or believed to have a TF prevalence of greater than 10% in children aged 1–9 years, for three years MDA with azithromycin donated through the ITI.

The data illustrated potential problems with the strategy of initiating MDA based on a 10% TF prevalence cut-off and continuing for three years. The 10% cut-off was decided by expert opinion, and was not evidence-based. The relationship between TF and ocular *Ct* infection is recognised to be inconsistent, especially in low prevalence settings and in communities that have received mass treatment. [9], [10], [11], [12], [13], [14], [15] In particular, TF is often found in the absence or near absence of infection. Thus, reliance on clinical signs for treatment decisions is likely to result in many communities in places like The Gambia being unnecessarily treated. Furthermore, in some communities, including the Jareng cluster of villages in The Gambia, [16], [17] a single round of mass treatment was found to cause a profound and sustained reduction in the prevalence of infection (not necessarily accompanied by a parallel reduction in, or absence of, TF), suggesting that in some settings it may not be necessary to continue MDA for three annual rounds.

The WHO further recommended that programmes should aim for a mass treatment coverage of at least 80% [4] on the basis that high treatment coverage will reduce re-emergence of infection in mass treated communities. Whether the extra programme effort involved in enhancing coverage further by, for example, spending extra time in targeted communities is worthwhile (particularly if, as in The Gambia, very few individuals are infected) is unknown. The Partnership for the Rapid Elimination of Trachoma (PRET) study therefore aimed to generate data that would be used to accelerate the trajectory towards trachoma elimination by addressing the role and potential benefits of enhanced coverage and of tests for infection in the control of active trachoma by MDA with azithromycin, in a randomized controlled trial. The trial was conducted in settings representative of different active trachoma prevalence: The Gambia (low), Tanzania (intermediate) and Niger (high). Here we report the main results of the PRET trial in The Gambia.

### Objectives and hypotheses

The trial aimed to compare the prevalence of TF and ocular *Ct* infection in 0–5 year olds three years post-baseline between 1) communities randomized to “standard” coverage versus communities randomised to “enhanced” coverage and 2) communities randomized to a laboratory-based strategy to discontinue treatment if the prevalence of either TF or *Ct* infection in 0–5 year-olds fell below 5% with 95% confidence (the stopping rule “SR”), compared to communities randomised to receive annual mass treatment for three years (the “3×” strategy).

The hypotheses were that the mean community prevalence of both TF and *Ct* infection in 0–5 year-olds would be reduced in the enhanced arm compared to the standard arm and would be non-inferior in the SR arm compared to the 3× arm.

## Methods

### Ethical approval

Ethical approval was obtained from the London School of Hygiene & Tropical Medicine (LSHTM), UK, Ethics Committee, and The Gambia government/Medical Research Council (MRC) Joint Ethics Committee, The Gambia (SCC 1107v2). Informed oral consent was obtained from the village leaders and informed written (thumbprint or signature) consent from each child's guardian at the time of examination. The informed written consent was additionally verified and signed by an independent witness.

### Study design

Details of the PRET study design have been published elsewhere [18], [19]. For census purposes, The Gambia is divided into geographically defined census enumeration areas (EAs) containing 600–800 people, which consist, variably, of one medium-sized village, several small villages, or a segment of a large village. The EA was the unit of randomization and implementation in PRET The Gambia. The study took place in four districts: Foni Bintang, Foni Kansala, Lower Baddibu and Central Baddibu (**Figure 2**), which had been earmarked for three years MDA in the NEHP's plan on the basis of an assessed or presumptive TF prevalence of ≥10%, and which contained 25, 17, 30 and 30 EAs, respectively. All EAs in these districts were eligible for inclusion in the study.

### Sample size

We estimated the sample size requirements using simulation studies which assumed a non-inferiority design for each intervention strategy with non-inferiority limits of 8%, a standard deviation of 0.05 within each arm, a correlation of 0.5 between baseline and year three results, no interaction between intervention strategies and the alternative hypothesis for each comparison. Simulated data were used to estimate how often a statistical test would yield a rejection of the null hypothesis.48 EAs (12 per arm) provided greater than 80% power for each main effect (95% one-sided non-inferiority). A sample size of 100 children per community was chosen for sampling so that if no children had TF or infection, the upper 95% confidence bound was less than 5%.

### Randomization

All 102 EAs in the 4 districts were randomly assigned by the study statistician to one of the four study arms: 1) Standard-SR; 2) Standard-3×; 3) Enhanced-SR; 4) Enhanced-3× under the restriction that all EAs that represented segments of the same village were in the same randomization group and would receive the same combination of delivery strategies. The restriction process also aimed for balance of strategy by district and overall. From these EAs a random selection of 48 ‘study EAs’ for sampling was made such that 12 study EAs per arm and per district were selected (three EAs per arm per district) and such that each large settlement was represented by only one of its segment EAs selected at random. The participants and census, examination and treatment teams were unaware of which EAs were allocated to which coverage arm.

### Census

In each of the 48 EAs, a complete census of all households was carried out. The census was updated every six months immediately before each survey round and new households and new persons in existing households were recorded, with births, deaths, and households or persons who no longer resided in the community being noted. The census was used to randomly select children and to provide the basis for data on coverage of mass treatment.

### Surveys

Details of data collection for PRET in The Gambia have been described elsewhere [19] but are summarised here. Surveys were conducted at seven time points: at baseline (May2008) and at 6, 12, 18, 24, 30 and 36 months thereafter. All children aged 0–5 years were identified from the census in each EA at each time point. Eligible children were: resident in the village, did not have an ocular condition precluding examination or specimen collection, willing to have a sample taken, and had a guardian willing to provide consent. These children were identified by the field team and a random selection of 100 children, and 20 reserve children, was made in the field using a list of random numbers. If one or more selected children could not be examined, the first or subsequent reserve child for that village was examined instead.

Baseline, 12 month, and 24 month surveys were carried out prior to annual MDA rounds. The infection and disease results from the 6, 18, and 30 month surveys were to be used to determine if the annual MDA scheduled for the following year next year should be stopped, if the community was in the SR arm.

### Sampling

Children were examined at a central site within each EA. Written informed consent was obtained from the guardian prior to the start of the examination. The examiner and specimen custodian wore gloves and changed them to avoid field contamination. Both eyelids of each child were everted and the tarsal conjunctiva graded for signs of clinical trachoma, using the WHO simplified grading system [20] by a standardised trachoma grader. Two photographs were then taken of the right everted upper tarsal conjunctiva, and an ocular sample was taken using a Dacron swab. Samples were kept cool in the field and frozen within 10 hours. For five samples chosen at random in each group of 100 ocular samples, air controls were collected immediately after the ocular sampling by passing a swab 5 cm from the child's right eye five times.

### Sample processing

Samples were processed at the Medical Research Council (MRC) laboratories in The Gambia using the Amplicor kit (Roche Molecular Systems, Pleasanton, CA, USA). Manufacturer's instructions were followed except for sample extraction where a previously published protocol was employed. [21]. Two *Ct* positive and two *Ct* negative processing controls were run with each batch of specimens. Samples whose optical density (OD) values in valid runs were ≥0.8 A_450_ were counted as positive, and samples less than 0.2 A_450_ were negative. Samples for which the result was equivocal (≥0.2, <0.8) were tested again in duplicate. The sample was only considered positive if the OD of one of the retests was ≥0.8. If equivocal twice or subsequently unresolved, they were left as indeterminate in the analyses. Due to the expected low prevalence, samples were tested in pools of five, and samples from positive pools were then retested individually to identify the positive sample.

### Intervention

EAs allocated to standard treatment coverage were treated on one day only (usual NEHP practice), which was expected to result in coverage of 80–89% in 0–9 year olds. For enhanced coverage, each EA was visited on one extra occasion to treat people who had not been treated on the first visit. This was expected to achieve coverage of 90% or more in 0–9 year olds. EAs allocated to the 3× arms received the recommended yearly MDA for three years. EAs in the SR arms received yearly MDA only if warranted by the presence of both infection and disease in one or more of the 100 children surveyed six months previously. If an EA allocated to an SR arm contained no cases of either infection or disease MDA would cease for the EA and the village(s) it contained, but continuing surveys monitored for re-emergent disease and infection. In addition, a ‘district level’ stopping rule was applied which meant that if no cases of infection or disease were found within the SR EAs in the district, treatment would also stop in all the other non-study EAs in the district.

### Treatment

Treatment was distributed to all 102 EAs in all 4 districts at baseline (July–August 2008). At 12 and 24 months post-baseline, EAs assigned to the 3× arm were mass treated. Treatment was distributed by the NEHP as part of the programme's country-wide mass treatment campaign. A central treatment station was set up in the village, and individuals were identified through use of the census. Adults aged 14 years or above received 1 g of azithromycin, whereas height was used as a surrogate for weight to dose children on the basis of 20 mg/kg. Treatment was observed and the number of tablets or ml of suspension recorded. Tetracycline eye ointment was offered to pregnant women and infants aged under 6 months, and noted in the treatment record.. All individuals who presented for treatment were treated, even if the result was higher than expected coverage in the communities randomised to the standard arm. Coverage was calculated as the proportion of individuals listed in the census who were treated.

### Concealment and blinding

The survey teams did not have access to the coverage assignment of the communities. The NEHP treatment team were not part of the survey and were unaware of treatment allocation on the first day they treated an EA. Laboratory personnel were masked to EA, coverage and treatment allocation.. At time points after six months, concealment of stopping rule allocations from participants and treatment teams was not possible due to the design of the intervention. .

### Outcome measures

The primary outcome measures were the EA-level prevalence of TF and of *Ct* infection in 0–5 year olds at the 36 months survey.

### Harmonisation and quality control

A standardisation workshop attended by investigators and key field personnel for all three trial sites was conducted in Ethiopia. Training and standardisation were performed for clinical grading, photography, sample collection, study forms, and data entry. Trachoma assessment was monitored at each time- point using field photographs from The Gambia and other sites, at least 50 per grader. These were graded after each survey by a senior grader (RB) masked to the field grades and to the grades of the images by the field graders. Graders were required to maintain a Cohen's kappa statistic of at least 0.6 against the reference grader for TF, and if agreement fell below 0.6 for any survey, re-standardisation was instituted to bring agreement to acceptable levels. Field and laboratory contamination was monitored over time using the field “air” controls, as well as internal laboratory controls for each run. If any field control was positive, then immediate investigation was undertaken to determine the source of contamination, and specimens re-run if needed to confirm results. To assure the quality of the laboratory results, random samples of 20 Amplicor positive and negative samples were sent blind to the reference laboratory at the Molecular Biology Laboratory, University of California, San Francisco (UCSF), USA, with at least 90% concordance required between the Gambian and UCSF laboratories. At 24 and 36 month time points samples were also sent to the laboratory of Dr Charlotte Gaydos at Johns Hopkins University. Data quality, results of data entry edit rates, of monitoring for quality of trachoma assessment. and of laboratory assessment of infection, were presented to a yearly meeting of the Data and Safety Monitoring Committee.

### Data entry and cleaning

Data were entered into a customised database (MS Access v2007) developed at the Dana Center, Johns Hopkins University. Key fields were double-entered by a different entry clerk. In addition to an in-built query generation system in the Access database, bespoke data cleaning programs were developed in Stata, version 11(StataCorp LP, College Station, Texas USA).

### Statistical analysis

All analyses were carried out using Stata, version 12 (StataCorp LP, College Station, Texas USA). Baseline population characteristics were summarised at cluster (EA)-level and then summarised according to randomization group as the mean of cluster means (SD) and the median (range) of cluster medians for each characteristic (or attribute). Comparisons at baseline used the Kruskal-Wallis test for non-parametric testing across more than two groups. Mass treatment coverage was estimated at cluster level in both 0–9 year olds and all residents, as the number recorded as treated divided by the number of children present in the census immediately prior to treatment

#### Primary analysis

Community-level data for each outcome at year three were modelled using negative binomial regression to account for zero prevalence in a high proportion of clusters in the intention-to-treat (ITT) population. Models included fixed effect terms for coverage and frequency and adjusted for baseline cluster-level prevalence *a priori*. A frequency-coverage intervention interaction was tested using a likelihood ratio test (LRT) comparing adjusted models with and without the interaction term. The analysis plan stated inclusion of the interaction term for a LRT p≤0.1.

#### Secondary analysis

Cluster-level year three data were modeled using negative binomial regression in the pre-specified as-treated (AT) population. Instead of coverage allocation, percentage cluster-level coverage in the last treatment round was modeled as a continuous variable. In addition, a fixed effect was included for time in years since last mass treatment in that community.

#### Sensitivity analysis

ITT and AT analyses were repeated using random effects logistic regression of individual level data. A *priori* adjustments were made for baseline cluster-level prevalence of the outcome and district.

#### Geographical clustering

EAs were considered adjacent if they had a common boundary. Moran's I statistic [22] and the rank adjacency statistic D with adjustment for sparse data [23] were used to evaluate geographical clustering of infections

## Results

### Participation of communities

**Figure 1:**
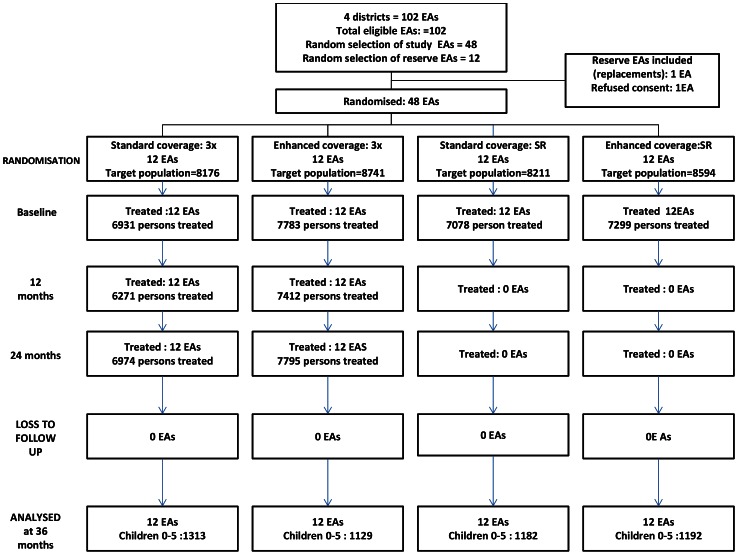
Flow of EAs and participants through the study.

Figure 1 shows the randomization scheme, and flow of EAs and participants through the study.

**Figure 2:**
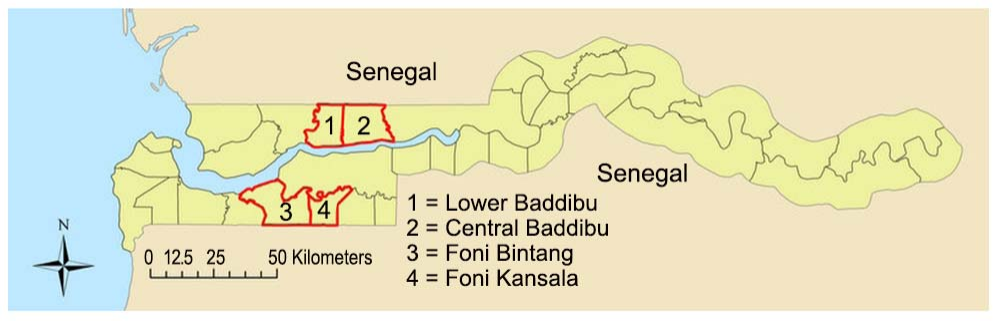
Location of PRET study districts within The Gambia.

### Baseline

A comparison of baseline characteristics of each group of communities showed no imbalances in population size, percentage of households with no latrine, percentage more than 30 minutes from water, or average education of head of household (Table 1). The baseline prevalence of TF and of infection with *Ct* was low and did not differ by study arm or allocation (Table 2).

**Table 1:** Baseline characteristics.

		Randomisation group			
Characteristic		Standard-3×	Enhanced-3×	Standard-SR	Enhanced-SR	p-value*
Number of clusters (EA)		12	12	12	12	
Number of households		729	839	801	753	
Total Population		8176	8741	8211	8504	
Total Population aged 0–5 years		1745	1864	1771	1774	
EA population size	Mean (SD)	681 (265.1)	728 (329.3)	684 (196.2)	709 (180.0)	
	Median (range)	617 (348–1255)	635 (453–1617)	664 (342–1118)	708 (327–952)	
Household head years of education:	Mean (SD)	0.8 (1.0)	0.4 (0.5)	0.5 (0.5)	0.5 (0.8)	0.838
	Median (range)	0.2 (0–2.9)	0.3 (0–1.4)	0.4 (0–1.6)	0.1 (0–2.7)	
Water more than 15 minutes away	Mean (SD)	13.3 (15.2)	10.7 (14.7)	14.6 (24.5)	10.6 (15.6)	0.937
	Median (range)	8.9 (0–50.0)	3.0 (0–40.4)	3.0 (0–85.7)	4.4 (0–52.6)	
Latrine access	Mean (SD)	91.1 (11.5)	88.9 (13.6)	89.2 (9.1)	94.8 (8.6)	0.623
	Median (range)	96.0 (66.7–100)	91.6 (59.0–100)	89.3 (74.5–100)	97.7 (74.4–100)	
Recall of community health education program	Mean (SD)	30.7 (21.3)	40.2 (31.7)	27.7 (18.9)	42.3 (22.9)	0.291
	Median (range)	22.1 (5.4–73.8)	27.0 (11.1–91.3)	23.3 (0–69.6)	43.1 (3.4–97.4)	

Data are summary statistics generated from cluster level prevalence, cluster level mean or cluster level median summary data.

* p-value from Kruskall-Wallis test across 4 allocation groups.

**Table 2:** Treatment coverage by randomisation group.

	Children under 10 years of age				All eligible individuals			
	Standard-3×	Standard-SR	Enhanced-3×	Enhanced-SR	Standard-3×	Standard- SR	Enhanced-3×	Enhanced-SR
**Baseline**								
Mean (SD)	87.7 (6.8)	87.2 (9.0)	90.2 (5.5)	90.0 (8.2)	85.5 (6.2)	85.7 (9.2)	86.8 (6.4)	87.0 (9.3)
Median (range)	90.0 (73.5–96.4)	92.0 (62.7–97.7)	91.4 (73.5–98.9)	92.6 (62.7–98.9)	87.2 (72.5–97.9)	86.0 (71.1–94.3)	88.6 (62.4–98.2)	90.1 (62.4–98.2)
**Year one** *								
Mean (SD)	84.8 (8.2)	-	89.5 (8.2)		82.5 (9.1)	-	87.8 (9.3)	
Median (range)	87.0 (68.9–96.8)	-	88.8 (68.9–99.4)		93.5 (64.3–96.1)	-	87.5 (64.3–99.2)	
**Year two** *								
Mean (SD)	88.8 (7.7)	-	91.3 (6.5)		87.4 (7.4)	-	89.8 (6.4)	
Median (range)	90.3 (69.6–99.1)	-	92.0 (69.6–99.4)		88.0 (73.6–99.5)	-	89.8 (73.6–99.5)	

Data are mean (SD) of cluster percentage coverage and median (range) of cluster percentage coverage.

* coverage estimation at years one and two based on 12 communities per coverage allocation.

### Coverage of MDA

**Figure 3:**
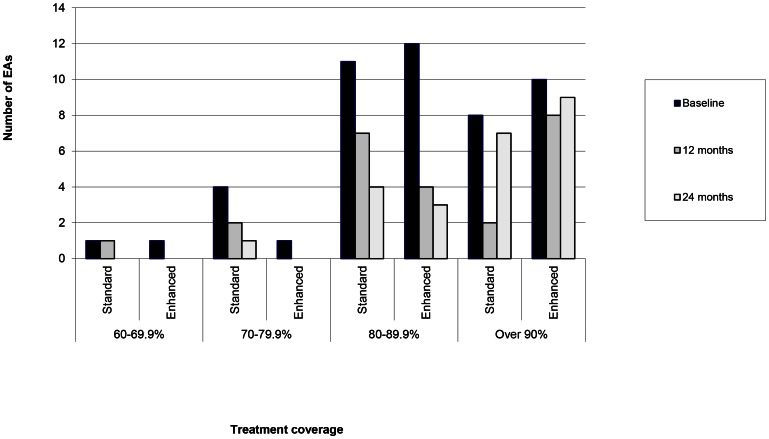
Treatment coverage in 0–9 s according to allocation and time point.

The treatment coverage of MDA at baseline was greater in the enhanced arms compared to the standard arms (Table 3). Not all of the communities in the standard arm reached 80% coverage of children under age ten and some exceeded 90% (Figure 3) Not all of the communities in the enhanced arms achieved the expected coverage of over 90% of children under age ten.

**Table 3:** District-level prevalence of trachoma outcomes in children 0–5.

	District			
	Foni Bintang	Foni Kansala	Lower Baddibu	Central Baddibu
**TF:**				
0	47/1212 (3.9: 2.9–5.1)	22/1259 (1.8: 1.1–2.6)	117/1274 (9.2: 7.7–10.9)	130/1280 (10.2: 8.6–11.9)
6	15/1200 (1.3: 0.7–2.1)	6/1269 (0.5: 0.2–1.0)	37/1262 (2.9: 2.1–4.0)	61/1317 (4.6: 3.6–5.9)
12	13/1163 (1.1: 0.6–1.9)	16/1209 (1.3: 0.8–2.1)	44/1255 (3.5: 2.6–4.7)	53/1178 (4.5: 3.4–5.8)
18	8/1148 (0.7: 0.3–1.4)	21/1238 (1.7: 1.1–2.6)	21/1274 (1.7: 1.0–2.5)	25/1257 (2.0: 1.3–2.9)
24	4/1131 (0.4: 0.1–0.9)	1/1186 (0.1: <0.1–0.5)	42/1237 (3.4: 2.5–4.6)	61/1219 (5.0: 3.8–6.4)
30	0/1188 (0: 0–0.3*)	2/1221 (0.2: <0.1–0.6)	63/1308 (4.8: 3.7–6.1)	75/1269 (5.9: 4.7–7.4)
36	2/1127 (0.2: <0.1–0.6)	3/1199 (0.3: 0.1–0.7)	47/1243 (3.8: 2.8–5.0)	80/1246 (6.4: 5.1–7.9)
**Ct:**				
0	21/1211 (1.7:1.1–2.6)	2/1259 (0.2: <0.1–0.6)	1/1274 (0.1: <0.1–0.4)	14/1279 (1.1: 0.6–1.8)
6	0/1186 (0: 0–0.3*)	2/1262 (0.2: <0.1–0.6)	0/1261 (0: 0–0.3*)	1/1313 (0.1: <0.1–0.4)
12	0/1163 (0: 0–0.3*)	0/1209 (0: 0–0.3*)	0/1255 (0: 0–0.3*)	0/1178 (0: 0–0.3*)
18	0/1146 (0: 0–0.3*)	0/1238 (0: 0–0.3*)	0/1270 (0: 0–0.3*)	0/1257 (0: 0–0.3*)
24	1/1119 (0.1: <0.1–0.5)	0/1186 (0: 0–0.3*)	2/1234 (0.2: <0.1–0.6)	0/1219 (0: 0–0.3*)
30	4/1173 (0.3: 0.1–0.9)	4/1216 (0.3: 0.1–0.8)	3/1301 (0.2: <0.1–0.7)	2/1255 (0.2: <0.1–0.6)
36	6/1128 (0.5: 0.2–1.2)	1/1198 (0.1: <0.1–0.5)	13/1241 (1.1: 0.6–1.8)	4/1235 (0.3: 0.1–0.8)

Data are number of case, n, out of number of children examined, N, (%: 95% CI).

Children with missing data excluded from district level summary analysis.

* one-sided 97.5% CI.

### Stopping rule

At the six month time point there was no *Ct* infection in either of the SR arms and the stopping rule was implemented, leading to all communities in the SR arms receiving no further rounds of MDA. The district level stopping rule, based on the same data, led to no further rounds of MDA being administered in any of the other 54 non-study EAs, so that within the 4 districts MDAs continued for two further rounds only in the 24 EAs belonging to the 3× arm. In these MDA rounds, treatment coverage in the enhanced-3× arm was consistently greater than that in the standard–WHO arm, but there were still communities in the standard-3× arm which achieved coverage outside the expected range of 80–89% and communities in the enhanced-3× arm which did not reach the expected coverage of ≥90% (Figure 3).

### TF

**Figure 4:**
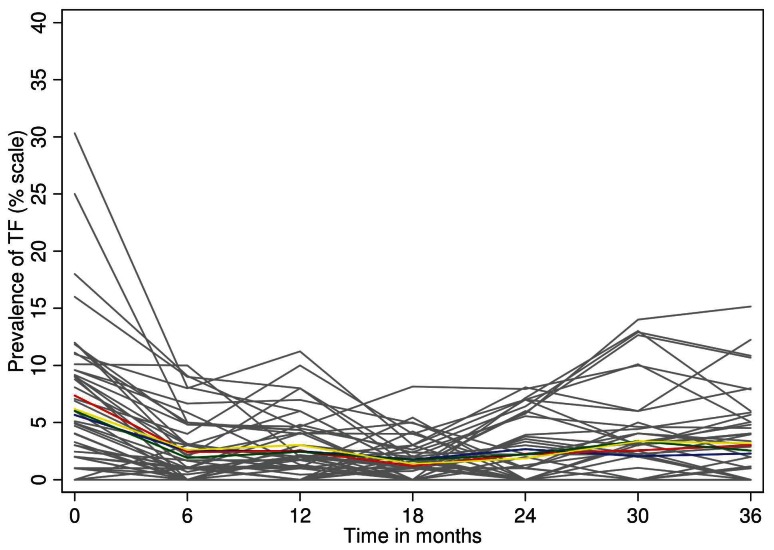
Cluster summarized mean percentage prevalence of TF by study arm (blue: standard 3×, red: , enhanced 3×, green: standard SR, yellow: enhanced SR and by individual EA within study arm(grey lines) at each time point.

Following the first MDA treatment there was a sustained reduction in TF prevalence throughout the study area, which was not different in any of the study arms or by allocation at any time point (Figure 4). At baseline, 44 of 48 EAs had non-zero TF prevalence, compared to 25 at 36 months. This resulted principally from the near-disappearance of TF in the Foni districts (Table 3) in contrast to an apparent reemergence in the Baddibu districts. At most time points there was no association between TF and the presence of *Ct* infection, either in individuals, EAs or districts (Table 3).

### Ct infection

**Figure 5:**
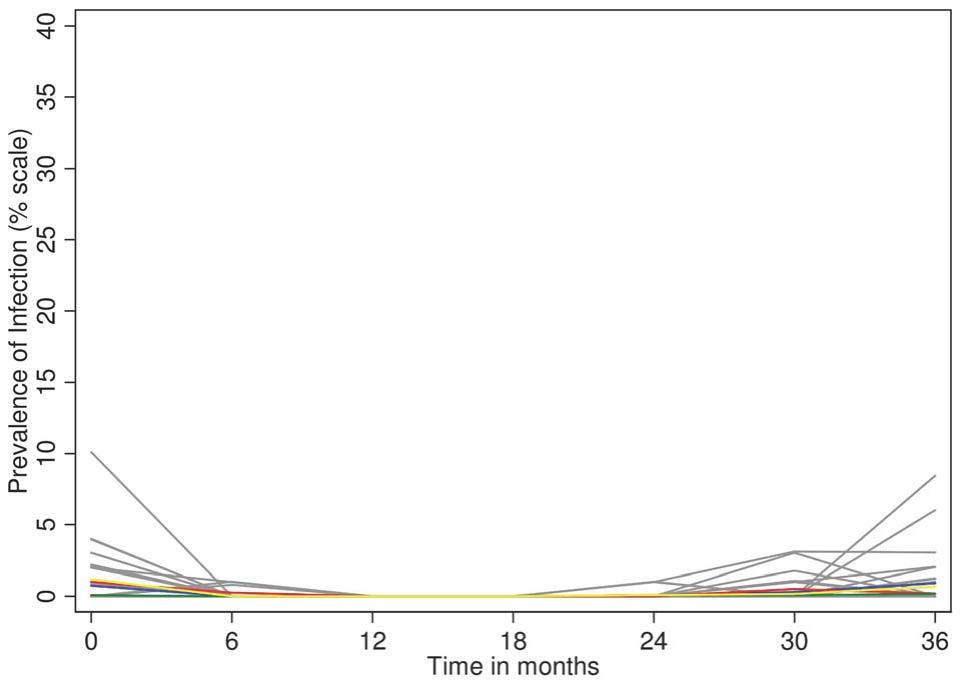
Cluster summarized mean percentage prevalence of *Ct* infection by study arm (blue: standard, 3× red: enhanced,3× green: standard, SR yellow: enhanced SR and by individual EA within study arm(grey lines) at each time point.

At each time point, the prevalence of *Ct* infection was similar between the study arms (Figure 5). At 12 and 18 months, no infection was detected anywhere in the study area, and at 36 months only 24 infections (0.5%) were found. There were fourteen EAs containing one or more *Ct* infections at baseline, and ten at 36 months.

### Outcomes

By 36 months, the TF prevalence across the study area had fallen from 6.5% to 2.8%. A model of TF prevalence at 36 months, adjusted for baseline prevalence and age of the sentinel group at 36 months, showed no effect of allocation to enhanced versus standard treatment, or of allocation to 3× with three annual MDAs versus stopping after one MDA in the SR arms (Table 4). The EA level prevalence of TF at baseline predicted the TF prevalence at 36 months. Residence in the Baddibu districts, which had the most TF at baseline, predicted the presence of TF at 36 months.

**Table 4:** Impact of mass treatment delivery strategies on *Ct* infection and TF at 36 months.

Intention to Treat Analysis	*Ct* infection		TF	
	Rate Ratio	95% CI	Rate Ratio	95% CI
Allocation to SR(one treatment) vs 3× (3 treatments)	0.78	0.14–4.49	1.17	0.65–1.53
Allocation to Enhanced versus Standard coverage	1.02	0.18–5.89	1.15	0.74–1.79
Cluster-level TF prevalence at baseline	0.92	0.46–1.87	1.14	1.06–1.23
**As-Treated Analysis**				
Frequency: Time in years since last mass treatment in community (3 vs 1 years)*	0.82	0.15–4.39	1.39	0.80–2.43
Percent coverage in community at last treatment round in 0–9 year olds†	1.02	0.90–1.15	1.02	0.98–1.05
Cluster-level TF prevalence at baseline	0.93	0.46–1.90	1.14	1.06–1.23

Effect estimates obtained from negative binomial model of cluster-level data.

* same as frequency (3× vs SR).

† modelled as continuous variable.

**Figure 6:**
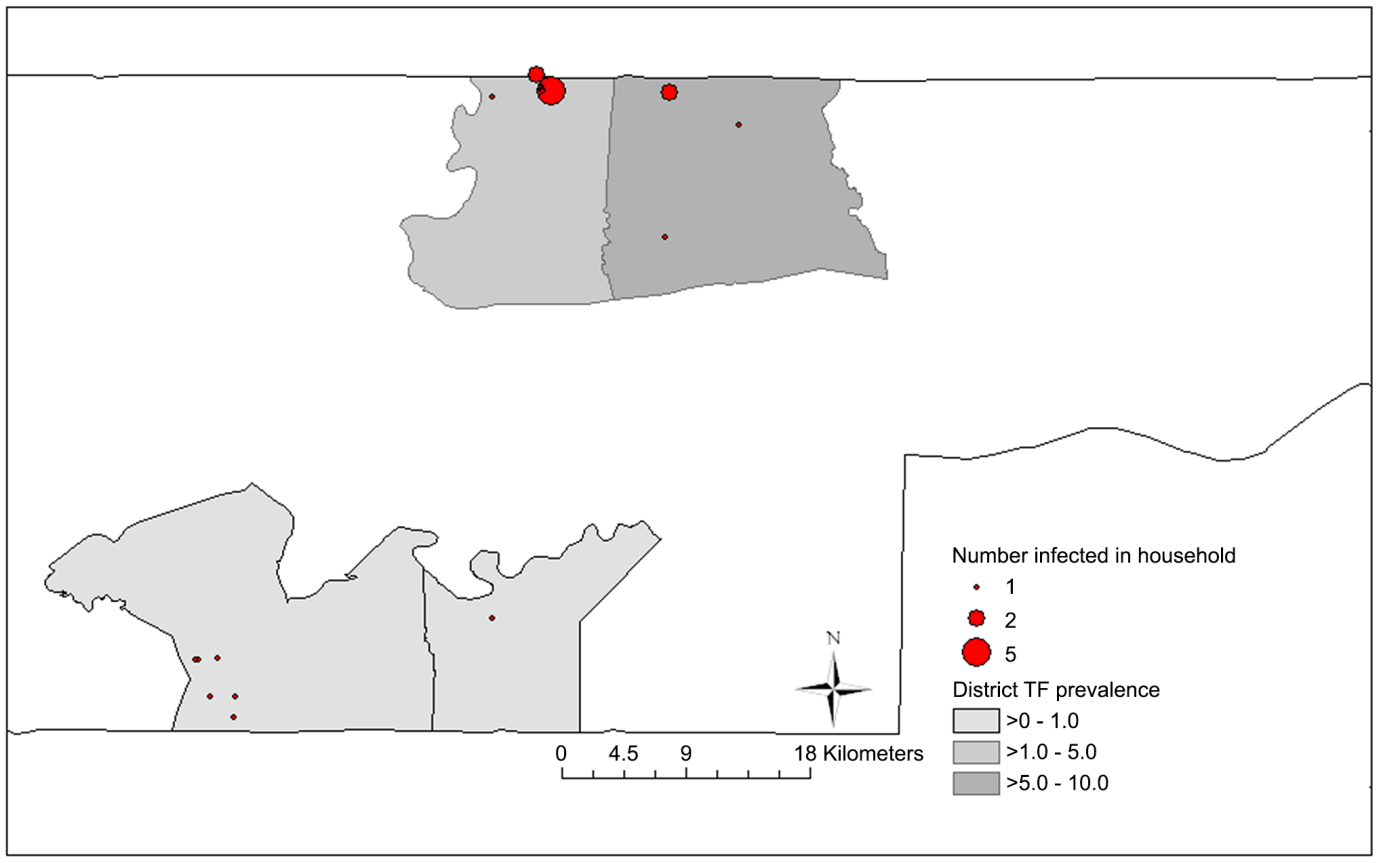
Geographical clustering of households containing infection at 36 month time point.

For *Ct* infection at 36 months, there were 24 infections (twelve in Standard-3× arm, eight in the Enhanced-SR arm and two each in the other arms) which also appeared geographically clustered. As per the analysis plan, the LRT used to test for evidence of a frequency-coverage interaction gave a result of p = 0.057, despite lack of evidence of any independent association between frequency and coverage strategies and infection. Rate ratios comparing each randomization group to the standard-3× group suggested lower point estimates for rates of infection for both enhanced groups and for the standard-SR group with no comparisons showing a significant difference. Twelve infections occurred among 124 children (9.6%) in three villages in Lower Baddibu near the Senegalese border (Figure 6) with the remaining twelve geographically dispersed among 4658 children (0.26%). The villages, although adjacent to each other, were in two separate EAs with different allocations: to standard-3× and enhanced-SR arms (as seen at the 36 month time point in Figure 5) Tests for spatial autocorrelation of 36 month EA level *Ct* infection prevalence suggested that infections were geographically clustered; (Moran's I = 0.228, z = 3.3, p<0.001; adjusted D 5.63, z = −2.2 p<0.05, with 64 adjacencies), and it is this clustering of re-emergent infection that accounts for the ‘interaction’. We have therefore not included the frequency-coverage interaction in the final regression model for infection (Table 4). In this model there was no evidence that either enhancing coverage or three rounds of annual treatment compared resulted in lower TF or *Ct* infection prevalence at 36 months.

The “as-treated” analysis (Table 4)also found no evidence that a lower TF or *Ct* infection prevalence at 36 months resulted either from increasing coverage above 80% in children, or from the last treatment in the community being one, rather than three years previously.

### Quality control: field grading

There were three graders. After baseline, some grader photo sets contained too little disease for proper assessment of agreement and photo sets taken in Tanzania or Niger were used to standardise at each time point. The graders maintained agreement kappas for TF above 0.6 with the senior grader at each time point (data not shown). Thus all graders passed the photo grading QA process.

### Quality control: laboratory

At the baseline, 6, 12, 18, 24, 30 and 36 month time points respectively, 280, 285, 278, 274, 235, 279, and 256 air controls were collected and processed. They were negative, except that *Ct* was detected on one air control at baseline as previously described [19]. Following the finding of no infection at all at 12 months, samples spiked with the Amplicor positive control in the field confirmed that positivity could still be recovered when it was expected to be present. The required concordance with the UCSF and JHU labs was achieved for all time-points (data not shown).

## Discussion

This study showed that, one year after a single round of azithromycin MDA, TF was reduced to low levels, and *Ct* infection became undetectable in sentinel communities across an area containing 67,000 people in 4 Gambian districts. These districts had already been selected for 3 rounds of MDA by the programme. In this setting there was no additional benefit of two further rounds of mass treatment. There was no difference in the prevalence of TF or of *Ct* infection at 36 months conferred by either enhancing treatment coverage through an extra visit, or by discontinuing MDA after one round (based on there being no infection in sentinel communities), rather than continuing for three. There were no baseline differences between arms for any of the assessed characteristics.

We sampled *Ct* infection and determined TF prevalence in children aged 0–5 years, as this group is most likely to harbour infection. This does introduce discrepancy with the WHO indicator of TF in 1–9 year olds. In clusters where all children 0–9 were examined at baseline the ratio between TF prevalence in 1–9 year olds and that in 0–5 year olds varied between 0.7 and 1.24 with a mean of 0.94 (data not shown). On this basis, we suggest that in these Gambian districts measuring TF in 0–5 year olds will represent a small underestimate of the WHO indicator. Similar observations have been made in Tanzania and Niger [24], [25].

Follow up was good, with all communities being retained in the trial at each time point. The clinical assessment of trachoma is always subject to inter-observer variation. In this study we attempted to minimise its influence with a harmonisation workshop and subsequent quality control procedures for TF grading involving regular assessment of grader performance using photographs, requiring kappa of above 0.6 with the senior grader. In a typical set of 50 photographs this represents fewer than six disagreements and thus some misclassification cannot be discounted. However further analysis of the grading quality control data found no evidence that systematic misclassification contributed to the reported decline in TF, as disagreements with the reference grader were generally equally common in both directions. As we report infections that are generally unrelated to clinical activity or prior treatment we considered contamination as a potential explanation, however analysis of the sequence and pattern of results in laboratory and the field suggested that positives occurred sporadically. The assessment of *Ct* infection was further validated through air swabs, glove precautions in the field, and exchange and repeat testing in other laboratories, the results of which were satisfactory. In addition, at time points when no infection was found, we were able to recover positivity from samples spiked in the field.

At the 36 month time point the prevalence of TF among 0–5 year olds in the study area was estimated to be 2.8%, in contrast to 6.5% at baseline. As can be seen in Figure 4 all of the decline in TF occurred after the first MDA, and was unaffected by subsequent MDAs. As shown in Table 4, TF in an EA was predicted by the baseline EA TF prevalence, indicating that the same EAs tended to have TF persistently. These observations may support the suggestion that a conjunctival follicular response (i.e. TF), once initiated in an individual, may be ‘recalled’ by exposure to other pathogens [26]. It is possible that older age groups might be a source of *Ct* exposure, but it seems unlikely that ongoing exposure to *Ct* can best explain either the persistence of TF in these communities, or its precipitate decline after only one round of MDA. The decline in TF to low levels after one round of MDA we observed in these districts suggests that there may be settings in which the recommendation to continue MDA, once initiated, for three rounds before resurvey is not appropriate. Here, a resurvey for TF after one MDA would have concluded that no further MDA was needed.

The prevalence of *Ct* infection was 0.8% in children aged 0–5 years at baseline, and it appeared sporadically distributed in a manner unrelated to TF in individuals, or TF prevalence in EAs or districts. No infection was found in any community at 12 or 18 months and there were only three infections among the 4638 children tested at the 24 month time point. Of the 24 infections in the study area at 36 months, nineteen were found in eight EAs in which infection was not previously demonstrable, and only five in two EAs which also contained participants with infection at 30 months(as illustrated by Figure 5). Twelve infections occurred in the adjacent border villages illustrated in Figure 6, where no infection had been found at any previous time point. Despite appearing in statistical analysis as an interaction between interventions, this clustered pattern of emergent infection is unlikely to be related to the MDA or coverage interventions implemented in the study, and is strongly suggestive of new infection introduced by recent contact with untreated communities. There are abundant untreated communities nearby, as every community we studied is within 15 km of the border with Senegal, where MDA for trachoma has not been used in the adjoining districts.

Whether reinfection from neighbouring countries or districts can lead to re-establishment of trachoma transmission and of the risk of blinding trachoma following local elimination is not clear. A previous study in The Gambia identified a large number of re-emergent infections in two communities associated with mass migration to a religious festival in Senegal, but the infections did not persist, suggesting that living standards had improved to such an extent that transmission of ocular *Ct* infection had become rare [16], [27]. On the other hand, failure to eliminate yaws by MDA has been attributed to a breakdown in surveillance once local elimination had been achieved; resulting in reintroduction of the disease once MDA had been discontinued [28]. The optimal strategy for post-elimination trachoma surveillance has not yet been defined. It is clear from the results of this and other studies that it is not sufficient to monitor the prevalence of clinical signs, as these may persist for years after infection has been eliminated, and are not well correlated with the presence of *Ct* infection.

Testing of communities for ocular *Ct* infection using nucleic acid amplification methods may be expensive, but the costs of such testing need to be balanced against the costs of unnecessary MDA rounds. Here, the use of a district level stopping rule requiring 95% confidence that the prevalence of *Ct* infection among 0–5 year olds in 24 sentinel communities after one round of MDA was less than 5%, allowed MDA to be discontinued across 4 districts containing 67,000 people. We do not know if 5% is the best cut-off for this type of decision, but when, as here, no infection was found in any of the sentinel communities after one round of MDA the decision that no further rounds of MDA are necessary is a straightforward one. Furthermore, we observe that if we had tested these communities and had the results available before the programme commenced MDA the ‘stopping rule’ would have been met and MDA might not have been started. Cheaper and simpler tests for ocular *Ct* infection would be useful to help guide treatment decisions and post-elimination surveillance. One candidate point-of-care test for infection has proved disappointing despite early encouraging results [29], [30] and there is little prospect of this, or a similar test becoming widely available. A serological test could potentially be used to monitor transmission if young children were tested, a strategy that has been used to document declines in malaria transmission in African communities [31].

The study team succeeded in improving treatment coverage through the strategy of making a second visit to each community. On average this increased coverage in under 10 s by slightly over 3% at each MDA round. The effect was smaller at baseline, when distribution coincided with early rains and agricultural activity, and at 24 months when the coverage achieved in one day was already above 90%. In this study, there was no evidence that this extra effort was worthwhile. Elsewhere, studies have shown that reinfection occurs despite high treatment coverage [32], [33]. In The Gambia, as in other settings [34], treatment coverage clustered by household, or alternatively the decision not to participate in MDA was taken at household level, but there was no evidence that households that did not participate had more disease or infection than those that did.

Worldwide, the ITI estimates that there are 216 districts with an estimated TF prevalence of 5–10% , containing over 40 million people (Rebecca Mann, Jennifer L Smith personal communications) [35], [36]. Many other districts may reach this prevalence range following intervention. Districts in which TF prevalence is between 5 and 10% of 1–9 year olds present a dilemma for trachoma control programmes, as the recommendation to re-survey community by community is considered expensive and impractical, and has not been endorsed by the donation programme. Here the 48 study EAs contained 115 village communities, whose baseline TF prevalence in 0–5 year olds varied from nothing to over 30%. As Table 5 shows, in 23(20%) of these villages TF prevalence was over 10% of 0–5 s, whereas 52(45%) contained no cases of TF at all in this age group. In this setting a single round of MDA reduced TF below the 5% elimination threshold.

**Table 5:** Baseline TF prevalences in children 0–5 y in 115 villages by district.

	District			
	Foni Bintang	Foni Kansala	Lower Baddibu	Central Baddibu
**TF prevalence 0–5 y**				
0	15	32	2	3
<5%	4	5	4	2
5–9.9%	5	3	5	8
10–19.9%	3	4	5	4
Above 20%	1	0	1	5

Data are number of villages with TF in each prevalence interval.

### Conclusion

In this study, where the baseline TF prevalence in an area containing 67,000 people was 6.5% in 0–5 year olds, a single round of MDA reduced TF below the elimination threshold, and a test for infection was applied to demonstrate that further rounds of MDA were redundant. Our study suggests that countries should consider treating such districts with a single high coverage round of MDA. It may however be difficult to distinguish these from other settings where treatment should continue without testing sentinel communities for *Ct* infection after treatment. The study further suggests that, following the MDAs in its national plan The Gambia has now reached the elimination target for active trachoma: the TF prevalence in 1–9 s may now be below the 5% threshold. This represents a significant achievement by the Gambian National Eye Health Programme.

## Supporting Information

Table S1 **CONSORT checklist**
(DOC)

Table S2 **Excel file indicating prevalence of ocular** ***Chlamydia trachomatis (Ct)*** **infection in each cluster at each time point.**
(XLSX)

Table S3 **Excel file indicating prevalence of TF in each cluster at each time point.**
(XLSX)
