# Correction: Mass Treatment with Azithromycin for Trachoma: When Is One Round Enough? Results from the PRET Trial in The Gambia

**DOI:** 10.1371/annotation/0bae8b34-5ae7-4044-a071-8d88d520a01b

**Published:** 2013-06-14

**Authors:** Emma M. Harding-Esch, Ansumana Sillah, Tansy Edwards, Sarah E. Burr, John D. Hart, Hassan Joof, Mass Laye, Pateh Makalo, Ahmed Manjang, Sandra Molina, Isatou Sarr-Sissoho, Thomas C. Quinn, Tom Lietman, Martin J. Holland, David Mabey, Sheila K. West, Robin Bailey, on behalf of the Partnership for Rapid Elimination of Trachoma (PRET) study group

The clinical trial registry information was accidentally omitted from this paper. It is registered at clinicaltrials.gov under the registration number NCT00792922. 

